# Kidney transplantation from a living donor to a mentally disabled recipient with bilateral angiomyolipomas—A case report

**DOI:** 10.1016/j.ijscr.2018.11.051

**Published:** 2018-11-24

**Authors:** Željko Vidas, Franjo Jurenec, Eva Lovrić, Marko Samardžija

**Affiliations:** a Department of Urology, Faculty of Medicine, Josip Juraj Strossmayer University of Osijek, Osijek, Croatia; b Department of Pathology, Merkur University Hospital, Zagreb, Croatia; c Clinic for Obstetrics and Reproduction, Faculty of Veterinary Medicine, University of Zagreb, Croatia

**Keywords:** Kidney transplantation, Mentally disabled transplant recipient, Living related donors, Angiomyolipoma

## Abstract

**Introduction:**

Many centers do not perform transplantation in mentally disabled people. Our patient with progressive psychomotor developmental delay had bilateral angiomyolipomas.

**Presentation of the case:**

Three years ago she underwent a right nephrectomy for massive spontaneous hemorrhage. The left kidney had a large, well-vascularized angiomyolipoma ready at any moment to bleed spontaneously was functioning normally. Two renal transplantation centers in Croatia refused to transplant from the patient's donor mother. The transplantation team had concerns whether to transplant a kidney to a person unable to care for herself, about who would take complete care of the patient, including regular immunosuppressive therapy, and whether it was ethically justified to explant a functioning kidney, although affected by angiomyolipomas, from a patient who required no renal replacement therapy at the time.

**Conclusion:**

We presented a successful kidney transplant in a mentally disabled person, clinical and ethical justifications for such a procedure, and a four-year post-transplant evaluation. Furthermore, in our opinion, renal transplantation in the mentally challenged needs to be referred to in literature exclusively as a relative contraindication instead of an absolute one, as has been practiced to date. This would facilitate transplantation teams deciding on kidney transplantation in mentally incapacitated individuals.

## Highlights

• Successful Transplantation of a Kidney from a Living Donor.
• Mentally Disabled Recipient Following Unilateral Nephrectomy.
• Massive Hemorrhage and Imminent Hemorrhage in the Normally Functioning Kidney.

## 1 Introduction

Kidney transplantation is now the globally accepted best treatment of patients with end-stage renal disease [[1], [2], [3]]. Although all patients are entitled to this kind of treatment and in spite of the provisions of law, treating mentally disabled persons by transplantation encounters resistance in many renal transplantation centers, partly because severe psychiatric illness and significant mental retardation are reported as being absolute contraindications for kidney transplantation [4,5]. The question arises as to what treatment should be used in patients with mental disablement, epilepsy and tumorous renal malformations [6].

Tuberous sclerosis is a rare autosomal dominant hereditary disease occurring due to a chromosome 9q34 or, in some families, chromosome 16 defect that causes mental retardation [7]. Multiple nodular tumors are composed of abnormal neuronal and glial, occasionally calcified, cells found in the cortex and in other parts of the brain. Progressive mental retardation and epileptic seizures develop in early childhood and are associated with a range of skin changes that resemble white spots and angiofibroma-adenoma sebaceum in a butterfly pattern on the cheeks, chin and forehead as the clinical hallmark of the disease [8]. With the progression of the disease angiomyolipomas, tumorous malformations, may occur in the kidneys, liver, adrenal gland and pancreas. Mentally disabled patients with epilepsy and such tumors rarely live longer than 30 years. Transplantation in a mentally disabled person is not a surgical problem; it is a problem because postoperative care is risky and complex [9].

## 2 Presentation of the case

The work has been reported in line with the SCARE criteria [10]. A young girl, born in 1984, was admitted for work-up and opinion on the feasibility of kidney transplantation in 2006 [11,12]. The patient was diagnosed with developmental delay, including with mental retardation and epilepsy in her early childhood. At the age of 4.5 months she was first hospitalized for seizures, diagnosed with West’s syndrome, and recommended antiepileptic therapy. A diagnosis of tuberous sclerosis was made in 1992. MRI of the brain showed visible native as well as post contract MRI of the brain showed typical changes characteristics for tuberous sclerosis with multiple hematomas cortically, subependimally, peri- and intra-ventriculary. Because of the arrest of psychomotor development, the patient was under the care of rehabilitation institutions. In July 2003 she was admitted to the urology department of another hospital for angiomyolipomas of both kidneys. Several days later an urgent nephrectomy of the right kidney with massive blood replacement was performed for massive spontaneous bleeding from the angiomyolipoma. Hemorrhages from the left renal angiomyolipoma, which continued to grow, occurred on several occasions afterwards and were treated by blood transfusion. The left kidney was therefore functioning with angiolipomatous lesions and the patient was at a high risk of spontaneous hemorrhage and bleeding to death.

The patient's mother volunteered to donate a kidney [13]. The standard pre-transplant workup protocol revealed no contraindications for organ donation. Tissue typing showed negative lymphocytotoxic antibodies, HLA phenotypes A2, A25, B35, B51, DR4, DR11 and DQ3. Both the recipient and the donor were of 0 Rh positive blood type [14,15], and MSCT angiography showed one artery per kidney. Regarding family genetics TSC1 and TSC2 genes mutations was not find. Two renal transplantation centers in the Republic of Croatia refused to perform the transplantation, although the patient's mother wished to donate a kidney.

The transplantation team had the following concerns: first, a dilemma whether to transplant a kidney to a person unable to care for herself; second, who would continue taking complete care of the patient, including administering regular immunosuppressive therapy; and, third, whether to explant a kidney that was functioning, although affected by angiomyolipomas, from a patient who at the time required no renal replacement therapy, was ethically justified. Because of the relatively high risk of severe blood loss and death, as well as the patient mother's big motivation to donate the organ, we decided to perform a transplantation and carried out the pre-transplant work-up. After undergoing a work-up at the Department of Nephrology, the patient was transferred to the Urology Division of the Department of Surgery on January 9, 2006.

The findings of the preoperative workup performed on admission were within normal ranges: leukocyte count 5.09 × 10^9^/L, red blood cell count 9.93 × 10^12^/L, hemoglobin 114 g/L, hematocrit 0.37 L/L, potassium 4.1 mmol/L, sodium 140 mmol/L, chloride 104 mmol/L, blood glucose 5.7 mmol/L, urea 4.0 mmol/L, creatinine 111 mmol/L, and C-reactive protein 2.0 mg/L. Abdominal and pelvic CT scans demonstrated a large angiomyolipoma filling the entire left retroperitoneal space up to the entrance of the small pelvis, invading the renal parenchyma, compressing the pyelocaliceal system and enclosing the left ureter. Contrast-enhanced scan did not reveal extravasations, hemorrhages nor hematomas.

One transplantation team used a transabdominal approach to perform a left nephrectomy in the recipient via median laparotomy. Pathohistology findings showed adipose tissue, smooth muscle component, thick walled blood vessels and abnormal tumor vessels. Also, we did not find any elements of malignant alteration. The other team simultaneously explanted the donor's kidney using a lumbar approach. The explanted donor kidney was then implanted into the recipient’s left iliac fossa. Cold ischemia time was 58 min and warm ischemia time was 35 min. The arterial anastomosis was sutured end-to-side to the external iliac artery using 6-0 prolen and the vein was sutured end-to-side to the external iliac vein by 5-0 prolen [16,17]. The ureter was sutured to the urinary bladder using the Lich-Gregoir technique with a double JJ stent insertion [18].

After the surgery, the patient was transferred to the intensive care unit (ICU) and stayed intubated and mechanically ventilated until the following morning. Laboratory test results were satisfactory, except for the C-reactive protein, which was 85 mg/L. Immunosuppression was continued (cyclosporine, mycophenolate mofetil, corticosteroids) with antibiotic and antifungal protection, H2 blockers, and *Pneumocystis carinii* prophylaxis [19,20]. The patient received immunosuppressive therapy, anticoagulant therapy, antileptics, antipsychotics, antibiotics and other support therapy if needed. Further, the patient was circulatory stable and with profuse diuresis. Volume replacement with colloids and crystalloids was carried out using analgesic and sedative medications. The patient was extubated on her second day at the ICU. The kidney ultrasound was satisfactory, with the correction of anemia; CMV prevention was carried out and antiepileptic drugs were given per os. The second day after the transplantation laboratory findings were as follows: blood glucose 7.8 mmol/L, urea 5.6 mmol/L, creatinine 131 mmol/L, potassium 4.4 mmol/L, sodium 146 mmol/L, chlorides 118 mmol/L, calcium 1.26 mmol/L, and magnesium 0.46 mmol/L. The patient gradually recovered at the ICU and was transferred to the nephrology division after six days.

The patient visited another transplantation center in her town of residence for follow-up examinations and our center for annual check-ups. Four years after the transplantation renal function was normal and the patient’s parents are taking care to adhere to immunosuppressive and other therapies. The ultrasound of the kidney graft four years after transplantation showed the kidney size was 127 mm, the width of parenchyma being approximately 16 mm, and that of the cortex 7–9 mm. The cortico-medullar margin was clearly visible, parenchyma was of normally echogenic structure and there were no signs of concretions. The canal system was not significantly dilated, with the pyelon transverse diameter being about 18 mm. The ultrasound also showed no perirenal collections.

## 3 Discussion

The successful transplantation and a functioning kidney are the best evidence that the decision to perform the surgery was correct. The first two concerns about whether to transplant a kidney to a mentally disabled person unable to care for herself and about further care of the patient were successfully resolved. Although many centers do not accept kidney transplantation in mentally disabled patients, it needs to be pointed out that everyone has an equal right to treatment. If a mentally disabled person requires dialysis, taking care of regular immunosuppressive therapy after transplantation is much more feasible to guardians than taking an uncooperative patient to hemodialysis two or three times a week.

**Fig. 1:**
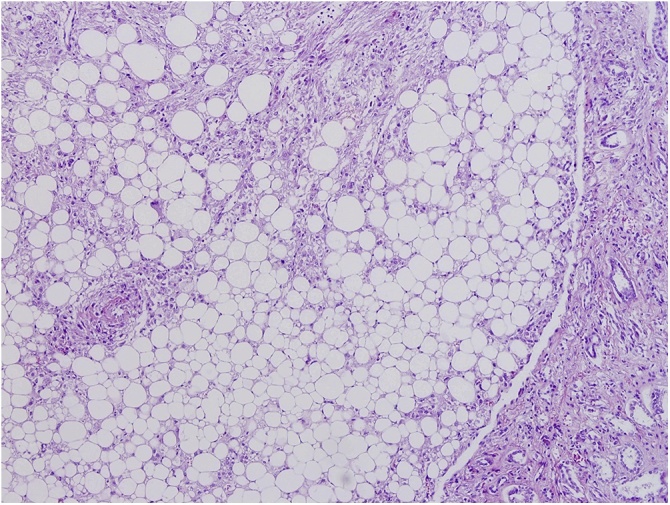
All three components of the tumor and normal renal tissue peripherally (HE; ×10).

**Fig. 2:**
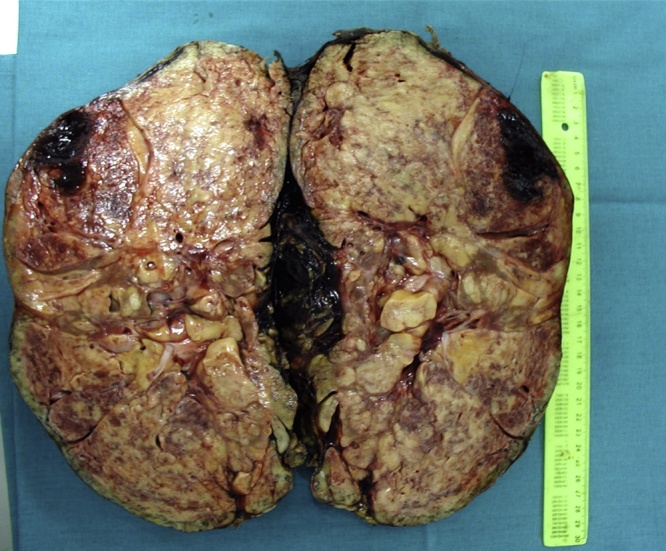
Cut surface of the kidney diffusely invaded by a yellowish-rosy tumor tissue and with areas of hemorrhage.

**Fig. 3:**
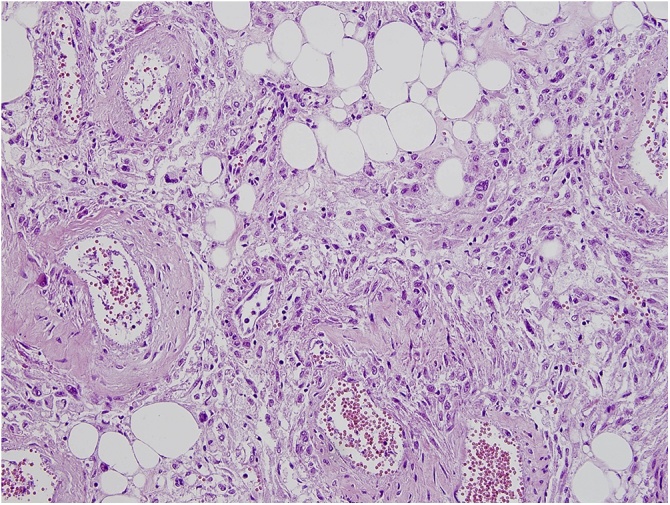
All three components of the tumor: fatty tissue, irregular muscle cells and blood vessels with thick, hyalinized walls (HE; ×20).

The macroscopy and pathohistology of the removed kidney answered our third concern as to whether a still functioning angiomyolipomatous kidney should be removed and what the risk of spontaneous bleeding was. The tumor size was 25 × 16 × 10 cm (Fig. 1). The entire parenchyma was invaded with yellowish-rosy tumor tissue with areas of hemorrhage, and the canal system was passable, but and compressed (Fig. 2, Fig. 3). The tumor was histologically composed of mature fatty tissue, blood vessels with thickened walls and radially distributed spindle-shaped smooth muscle cells. Muscle cell bundles and fat cells infiltrated the renal parenchyma in two areas, in the fibrous renal capsule, and in the surrounding fatty tissue. The pathophysiology made it clear that it was only a matter of time until a massive hemorrhage occurred and the patient was vitally compromised. Kidney transplantation from a living donor is a complex process, and specific cases such as this one sometimes present ethical and medical dilemmas that must be carefully considered to reach a proper decision about the treatment method.

## 4 Conclusion

In conclusion, by performing this procedure and by following-up the patient with an adequately functioning kidney transplant on a regular and long-term basis supported by family care for the patient's compliance with immunosuppressive and other therapy, we have demonstrated that renal transplantation in mentally insufficient patients should become a routine practice rather than constitute the controversy among transplantation professionals. Furthermore, in our opinion, renal transplantation in the mentally challenged needs to be referred to in the literature exclusively as a relative contraindication instead of an absolute one as has been practiced to date. This would facilitate transplantation teams deciding on kidney transplantation in mentally incapacitated individuals.

## Conflict of interest

The authors of this manuscript have no conflict of interest to disclose as described by International Journal of Surgery Case Reports. The Results presented in this paper have not been published previously in whole or part.

## Funding

None source of funding.

## Ethical approval

The manuscript is not a clinical trial, but the surgical procedure performed on the patient. In accordance with that we didn’t need any Ethical Committee approval.

## Consent

Written informed consent was obtained from the patient for publication of this case report and accompanying images. A copy of the written consent is available for review by the Editor-in-Chief of this journal on request.

## Author contribution

Željko Vidas, surgery procedures, the conception and design of the study, drafting the article and revising it critically for important intellectual content, final approval of the version to be submitted.

Franjo Jurenec, surgery procedures, the conception and design of the study, drafting the article and revising it critically for important intellectual content, final approval of the version to be submitted.

Eva Lovrić, histopathology analyses, the conception and design of the study, drafting the article and revising it critically for important intellectual content, final approval of the version to be submitted.

Marko Samardžija, the conception and design of the study, drafting the article and revising it critically for important intellectual content, final approval of the version to be submitted.

## Registration of research studies

The manuscript is not a clinical trial, but the surgical procedure performed on the patient. In accordance with that we didn’t need any Ethical Committee approval.

## Guarantor

Željko Vidas, DM, PhD, Assistant Professor.

## Provenance and peer review

Not commissioned, peer reviewed.
